# Associations of atrophic gastritis and proton-pump inhibitor drug use with vitamin B-12 status, and the impact of fortified foods, in older adults

**DOI:** 10.1093/ajcn/nqab193

**Published:** 2021-06-16

**Authors:** Kirsty M Porter, Leane Hoey, Catherine F Hughes, Mary Ward, Michelle Clements, Jj Strain, Conal Cunningham, Miriam C Casey, Fergal Tracey, Maurice O'Kane, Kristina Pentieva, Liadhan McAnena, Kevin McCarroll, Eamon Laird, Anne M Molloy, Helene McNulty

**Affiliations:** Nutrition Innovation Centre for Food and Health (NICHE), School of Biomedical Sciences, Ulster University, Coleraine, Northern Ireland, United Kingdom; Nutrition Innovation Centre for Food and Health (NICHE), School of Biomedical Sciences, Ulster University, Coleraine, Northern Ireland, United Kingdom; Nutrition Innovation Centre for Food and Health (NICHE), School of Biomedical Sciences, Ulster University, Coleraine, Northern Ireland, United Kingdom; Nutrition Innovation Centre for Food and Health (NICHE), School of Biomedical Sciences, Ulster University, Coleraine, Northern Ireland, United Kingdom; Nutrition Innovation Centre for Food and Health (NICHE), School of Biomedical Sciences, Ulster University, Coleraine, Northern Ireland, United Kingdom; Nutrition Innovation Centre for Food and Health (NICHE), School of Biomedical Sciences, Ulster University, Coleraine, Northern Ireland, United Kingdom; Mercer's Institute for Research on Ageing, St James's Hospital, Dublin, Ireland; Mercer's Institute for Research on Ageing, St James's Hospital, Dublin, Ireland; Causeway Hospital, Northern Health and Social Care Trust, Coleraine, Northern Ireland, United Kingdom; Clinical Chemistry Laboratory, Western Health and Social Care Trust, Altnagelvin Hospital, Londonderry, Northern Ireland, United Kingdom; Nutrition Innovation Centre for Food and Health (NICHE), School of Biomedical Sciences, Ulster University, Coleraine, Northern Ireland, United Kingdom; Nutrition Innovation Centre for Food and Health (NICHE), School of Biomedical Sciences, Ulster University, Coleraine, Northern Ireland, United Kingdom; Mercer's Institute for Research on Ageing, St James's Hospital, Dublin, Ireland; School of Medicine, Trinity College Dublin, Dublin, Ireland; School of Medicine, Trinity College Dublin, Dublin, Ireland; Nutrition Innovation Centre for Food and Health (NICHE), School of Biomedical Sciences, Ulster University, Coleraine, Northern Ireland, United Kingdom

**Keywords:** vitamin B-12 biomarkers, atrophic gastritis, proton pump inhibitor drugs, fortified foods, food-bound malabsorption, hypochlorhydria, older adults

## Abstract

**Background:**

Atrophic gastritis (AG) and use of proton pump inhibitors (PPIs) result in gastric acid suppression that can impair the absorption of vitamin B-12 from foods. The crystalline vitamin B-12 form, found in fortified foods, does not require gastric acid for its absorption and could thus be beneficial for older adults with hypochlorhydria, but evidence is lacking.

**Objectives:**

To investigate associations of AG and PPI use with vitamin B-12 status, and the potential protective role of fortified foods, in older adults.

**Methods:**

Eligible participants (*n* = 3299) not using vitamin B-12 supplements were drawn from the Trinity-Ulster and Department of Agriculture cohort, a study of noninstitutionalized adults aged ≥60 y and recruited in 2008–2012. Vitamin B-12 status was measured using 4 biomarkers, and vitamin B-12 deficiency was defined as a combined indicator value < −0.5. A pepsinogen I:II ratio <3 was considered indicative of AG.

**Results:**

AG was identified in 15% of participants and associated with significantly lower serum total vitamin B-12 (*P* < 0.001) and plasma holotranscobalamin (holoTC; *P* < 0.001), and higher prevalence of vitamin B-12 deficiency (38%), compared with PPI users (21%) and controls (without AG and nonusers of PPIs; 15%; *P* < 0.001). PPI drugs were used (≥6 mo) by 37% of participants and were associated with lower holoTC concentrations, but only in participants taking higher doses (≥30 mg/d). Regular, compared with nonregular, consumption of fortified foods (i.e., ≥5 and 0–4 portions/wk, respectively) was associated with higher vitamin B-12 biomarkers in all participants, but inadequate to restore normal vitamin B-12 status in those with AG.

**Conclusions:**

Older adults who have AG and/or use higher doses of PPIs are more likely to have indicators of vitamin B-12 deficiency. Fortified foods, if consumed regularly, were associated with enhanced vitamin B-12 status, but higher levels of added vitamin B-12 than currently provided could be warranted to optimize status in people with AG.

## Introduction

Food-bound malabsorption is widely considered the main contributor to subclinical vitamin B-12 deficiency in older adults in high-income countries (1). Atrophic gastritis (AG) (2) and chronic use of acid suppression drugs such as proton pump inhibitors (PPIs) (3) are among the contributing factors to vitamin B-12 malabsorption. Vitamin B-12 deficiency is recognized as a global problem, although prevalence rates differ between and within countries, depending both on the biomarkers used, and cutoff values applied, to define deficiency (1, 4). Because low vitamin B-12 status is associated with higher risk of several age-related diseases (5, 6), maintaining optimal vitamin B-12 status is a public health priority.

The presence of AG increases with age and results in gastric acid suppression (hypochlorhydria), thus limiting vitamin B-12 absorption because gastric acid is essential for the release of vitamin B-12 from food proteins (2). Consequently, low vitamin B-12 status can arise in older adults, as evidenced by metabolic changes but often without the classical hematological or neurological signs of clinical deficiency. Although some previous studies have identified AG using serum pepsinogen, the reported prevalence rates in older adults vary greatly from 7% (7) to 32% (8, 9). Moreover, the condition is largely undiagnosed in older populations, prompting concern that led to recommendations in the United States for adults aged >50 y to consume most of their vitamin B-12 from fortified foods or supplements (10), on the basis that absorption of crystalline vitamin B-12 has no gastric acid requirement (5). There is, however, limited evidence to support this recommendation, because few previous studies have reported rates of AG in relation to vitamin B-12 status (8, 9) or addressed the role of crystalline vitamin B-12 in improving status (7, 11).

PPIs are gastric acid suppression drugs, commonly used to treat conditions that are caused (or exacerbated) by an overproduction of stomach acid (e.g., simple heartburn, gastroesophageal reflux disease, Zollinger–Ellison syndrome). By inducing hypochlorhydria, there is concern that these drugs, widely prescribed to older adults (12) and also available over the counter (13), could also contribute to food-bound vitamin B-12 malabsorption with long-term use (14) and ultimately vitamin B-12 deficiency in an already vulnerable group. The evidence to date is inconsistent, however, with some studies showing no association of PPIs with vitamin B-12 (15, 16) and others reporting an increased risk of vitamin B-12 deficiency with long-term PPI use (17–19).

A limited number of previous studies have examined vitamin B-12 status in relation to AG or PPI drug use and these typically used a single biomarker. Given the limitations of each of the direct [total vitamin B-12 and holotranscobalamin (holoTC)] and functional [homocysteine and methylmalonic acid (MMA)] biomarkers, there is general agreement that use of a sole vitamin B-12 biomarker should be avoided and ≥2 biomarkers used to diagnose deficiency (20–22). Furthermore, to our knowledge, the potential of vitamin B-12–fortified foods to protect against vitamin B-12 depletion caused by hypochlorhydria has not been considered. The aim, therefore, was to investigate associations of AG and PPI use with vitamin B-12 status, and the potential protective role of fortified foods, in older adults. We hypothesized that vitamin B-12–fortified foods would alleviate any adverse associations of AG and PPI use and vitamin B-12 status.

## Methods

### Study design and participants

This observational study involved analysis of data from the Trinity-Ulster and Department of Agriculture (TUDA) cohort (clinicaltrials.gov identifier: NCT02664584), comprised of 5186 community-dwelling older adults. As described elsewhere (23), study participants were recruited between 2008 and 2012 from general practice or hospital outpatient clinics in Northern Ireland (United Kingdom) and the Republic of Ireland using standardized protocols. Briefly, the inclusion criteria for the TUDA study were: born on the island of Ireland, aged ≥60 y, and without an existing diagnosis of dementia. A comprehensive health and lifestyle questionnaire was administered as part of the 90-min interview to capture medical and demographic details, medications, and vitamin supplement usage. Weight, height, waist, and hip measurements were recorded and blood pressure (millimeters mercury) was measured in accordance with standard operating procedures. Ethical approval was obtained from the Office for Research Ethics Committees Northern Ireland (ORECNI; ref. 08/NIR03/113), with corresponding approval from the Northern and Western Health and Social Care Trusts in Northern Ireland, and the Research Ethics Committee of St James's Hospital and The Adelaide and Meath Hospital, Dublin. All participants provided written informed consent at the time of recruitment.

**FIGURE 1:**
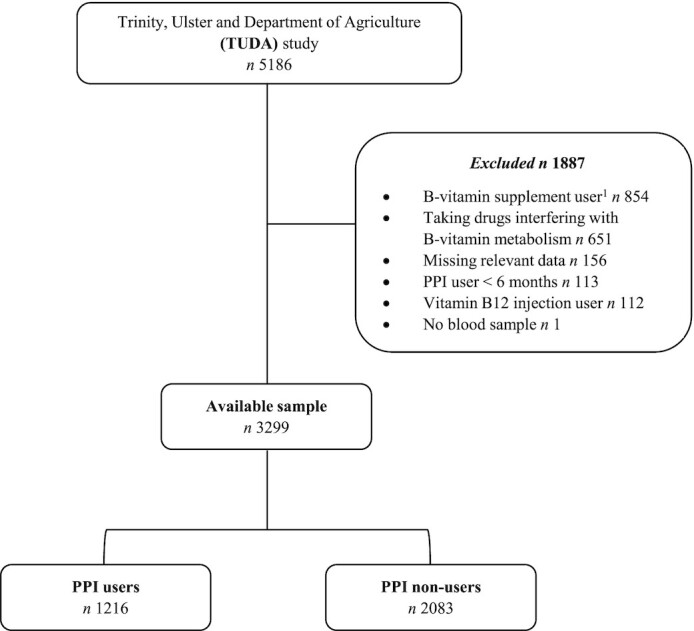
Flow diagram of eligible participants from the Trinity-Ulster and Department of Agriculture (TUDA) study. ^1^Based on self-reported supplement use and/or status of vitamin B-12 biomarkers. PPI, proton-pump inhibitors.

For this analysis, the following participants were excluded: those on vitamin B-12 injections (*n* = 112) and/or oral B-vitamin supplement users (*n* = 854); users of medication known to interfere with B-vitamin metabolism (e.g., metformin, anticonvulsants; *n* = 651); those taking PPIs <6 mo (*n* = 113), and those missing relevant data (*n* = 157), resulting in a final sample of 3299 participants for investigation (**Figure 1**).

### Fortified food intake assessment

Dietary information specifically focusing on habitual intake of foods fortified with vitamin B-12 was collected using a 7-item subsection completed as part of a larger FFQ used in the TUDA study, which had been previously validated for B-vitamin intakes against B-vitamin biomarkers (24). This subsection was comprised of 6 product categories known to be fortified with B-vitamins at the time of blood sampling. These product categories included ready-to-eat breakfast cereals, some fat spreads, and cereal snack bars, along with an “Other” option to capture information on fortified products that were consumed but not specified in the product categories in the FFQ. Brand names of fortified food products were collected by the researchers so that up-to-date details on relevant nutrient profiles could be verified. To examine the impact of vitamin B-12–fortified food consumption on vitamin B-12 biomarkers, participants were classified as nonregular (0–4 portions/wk) or regular (≥5 portions/wk) consumers of fortified foods. These categories were used in the current analysis based on our previous findings in the TUDA cohort where 4 categories of fortified foods were investigated, from 0 to >7 portions/wk. No significant difference in total vitamin B-12 concentrations was observed between participants identified as nonconsumers compared with those categorized as low consumers (1–4 portions/wk) of fortified foods, whereas ≥5 portions/wk were associated with higher biomarker status (23). One participant could not be classified as regards fortified food intake and is not included in this analysis.

### Blood sampling and laboratory analysis

All participants provided a nonfasting (50-mL) blood sample and all samples were processed within 4 h of collection. Analysis for routine clinical blood biochemistry profile was performed at the time of blood collection in participating hospital laboratories. Blood aliquots for all other biochemical measurements were stored at −70°C until batch analysis.

Serum pepsinogen I and pepsinogen II concentrations were measured using ELISA kits (US Epitope diagnostics) at Ulster University in nonusers of PPIs only; a ratio of pepsinogen I:II <3 was indicative of AG. The primary study outcome (vitamin B-12 status) was determined using 2 direct and 2 functional biomarkers. Serum total vitamin B-12 was measured by microbiological assay with *Lactobacillus leichmannii* (25) and holoTC by microparticle enzyme immunoassay (AxSym Active-B12; Axis Shield) (26). The functional biomarkers, MMA and plasma homocysteine, were measured by GC-MS with methylchloroformate derivatization and by fluorescence polarization immunoassay using the Abbot Imx Analyzer, respectively (Axis Shield) (27). MMA measurements were restricted to a subset of 1477 participants including all participants with either total vitamin B-12 ≤148 pmol/L or holoTC ≤30 pmol/L (815 samples in total) plus 598 random samples (selected using a random number generator) with both metabolites above these limits. Analysis of vitamin B-12 biomarkers was centralized in the Vitamin Research Laboratory at Trinity College Dublin (serum total vitamin B-12, holoTC, and plasma homocysteine) or at the University of Bergen, Norway (serum MMA). The secondary study outcome, a combined indicator of vitamin B-12 status, was calculated using serum total vitamin B-12, serum holoTC, and plasma homocysteine concentrations, age, and serum folate as described elsewhere (20). MMA was not included in this calculation because data were only available for 30% of participants, as noted above. A combined indicator value of vitamin B-12 status ≤ −0.5 was defined as deficient.

For all biomarkers except pepsinogen, samples were analyzed blind and in duplicate. Pepsinogen was analyzed blind in a single run. Quality controls (QCs) were provided by repeated analysis of pooled samples. Interassay CVs were as follows: serum total vitamin B-12 <10.9%; holoTC <11.1%; MMA <5.0%; plasma homocysteine <5.2%; pepsinogen I, 5.9% for QC1 and 9.0% for QC2, and pepsinogen II, 12.0% for QC1 and 18.2% for QC2. QC samples were supplied by the manufacturer of the ELISA kits for pepsinogen analysis.

### Statistical analysis

Statistical analysis was performed using SPSS software (Version 25.0; SPSS UK Ltd). Prior to analysis, variables were tested for normality and log-transformed as appropriate. Vitamin B-12 biomarkers and other relevant variables were compared among participants classified into 1 of 3 groups: PPI users, AG, or controls (i.e., not PPI users and pepsinogen I:II ratio ≥3). For numerical data, differences between groups were examined using either 1-factor between-groups ANOVA or ANCOVA after adjustment for age, sex, BMI, RBC folate, creatinine, fortified food consumption, and alcohol units per week with Bonferroni post hoc comparisons. For categorical data, differences between groups were examined using χ^2^ analysis. Correlations between age and vitamin B-12 biomarkers were performed by using Pearson correlation coefficients. For all analyses, *P* < 0.05 was considered statistically significant.

## Results

The identification of the sample for inclusion in this analysis from the TUDA study cohort is illustrated in Figure 1. Of the total cohort of 5186 TUDA participants, 3299 met the inclusion criteria for the current study. Characteristics of the participants in relation to PPI drug usage are shown in **Table 1**. Some 37% of participants reported taking PPI drugs (for a duration of ≥6 mo), with >90% of PPI users reporting a dose range of 20–40 mg/d. Compared with nonusers, PPI users were older, had higher BMI, greater alcohol intakes, and a higher prevalence of pre-existing cardiovascular conditions, whereas cholesterol concentrations and blood pressure were lower than in non-PPI users. AG was identified in 15% of PPI nonusers. People with compared to those without AG were significantly older (74.0 ± 7.8 y compared with 72.8 ± 8.0 y; *P* = 0.011) (not shown). The majority of the overall study cohort (71%) consumed foods fortified with vitamin B-12 at least once per week (Table 1).

**TABLE 1:** Characteristics of TUDA study participants in relation to PPI drug usage (*n* = 3299)^1^

	PPI nonusers	PPI users	
	*n* = 2083	*n* = 1216	*P* value^2^
General characteristics			
Age, y	73.0 (72.6, 73.3)	75.6 (75.1, 76.1)	<0.001
Sex, *n* (% male)	723 (35)	383 (32)	0.065
BMI, kg/m^2^	27.5 (27.3, 27.7)	28.1 (27.8, 28.4)	0.001
Waist/hip ratio, cm	0.91 (0.90, 0.91)	0.91 (0.91, 0.92)	0.056
Medical			
Atrophic gastritis, *n* (%)	317 (15)	—	—
Diabetes, *n* (%)	91 (4)	95 (8)	<0.001
Creatinine, µmol/L	83.0 (81.1, 84.1)	89.9 (88.4, 91.4)	<0.001
LDL cholesterol, mmol/L	2.52 (2.48, 2.56)	2.40 (2.35, 2.45)	<0.001
HDL cholesterol, mmol/L	1.51 (1.49, 1.53)	1.44 (1.42, 1.47)	<0.001
Triglycerides, mmol/L	1.56 (1.53, 1.60)	1.61 (1.56, 1.66)	0.089
Hyperlipidemia, *n* (%)	1032 (50)	677 (56)	0.001
Hypertensive, *n* (%)	1259 (61)	710 (59)	0.278
Systolic BP, mmHg	146.0 (145.1, 146.9)	144.2 (143.0, 145.4)	0.001
Diastolic BP, mmHg	79.1 (78.6, 79.6)	78.2 (77.5, 78.8)	0.009
Previous myocardial infarction, *n* (%)	152 (7)	167 (14)	<0.001
Previous stroke, *n* (%)	112 (5)	124 (10)	<0.001
Health and lifestyle			
Current smoker, *n* (%)	258 (12)	148 (12)	0.899
Alcohol,^3^ units/wk	8.1 (7.4, 8.7)	9.0 (8.0, 9.9)	0.011
Fortified food consumer,^4^*n* (%)	1491 (72)	848 (70)	0.278
Vitamin D supplement user, *n* (%)	894 (43)	561 (46)	0.175

1 Data presented are adjusted mean (95% CI) unless otherwise indicated. BP, blood pressure; PPI, proton-pump inhibitor; TUDA, Trinity-Ulster and Department of Agriculture.

2 *P* < 0.05; analysis via χ^2^ for categorical variables or ANCOVA for continuous variables (adjusted for age, sex, BMI) on log-transformed data as appropriate with Bonferroni post hoc tests.

3 Alcohol (units per week); 1 unit equates with 25 mL spirits, 220 mL beer, or 85 mL wine.

4 Participants who consumed foods fortified with vitamin B-12 at least once per week.

Age was inversely associated with total vitamin B-12 (*r* = −0.093, *P* < 0.001) and holoTC (*r* = −0.157, *P* < 0.001) concentrations and positively associated with homocysteine (*r* = 0.303, *P* < 0.001) and MMA concentrations (*r* = 0.139, *P* < 0.001; not shown). The associations of PPI usage and AG with vitamin B-12 biomarkers were examined (**Table 2**). AG was associated with significantly lower serum total vitamin B-12 (*P* < 0.001) and plasma holoTC concentrations (*P* < 0.001), and a higher prevalence of vitamin B-12 deficiency, compared with PPI users and controls [combined B12 (cB12) indicator; 38% compared with 21% compared with 15%; *P* < 0.001], after adjustment for age, sex, BMI, alcohol, fortified food consumption, creatinine, and RBC folate. MMA concentrations were also significantly higher in the AG group compared with PPI users and controls (adjusted means: 0.64; 95% CI: 0.54, 0.73; compared with 0.46; 95% CI: 0.40, 0.52; compared with 0.43; 95% CI: 0.48, 0.73; *P* < 0.001; not shown) when examined in a subset (30%) of the overall cohort with available MMA data. PPI medication use (≥6 mo) was associated with significantly lower holoTC concentrations (*P* < 0.001) compared with controls, but only in participants taking higher PPI doses (≥30 mg/d).

**TABLE 2:** Participant characteristics and concentrations of biomarkers of vitamin B-12 status with respect to PPI usage and atrophic gastritis (*n =* 3290)^1^

		PPI users^2^			
	Controls^3^	<30 mg/d	**≥**30 mg/d	Atrophic gastritis	
	*n* = 1766	*n =* 613	*n =* 594	*n =* 317	*P* value^4^
Age, y	72.8 (72.4, 73.2)^a^	74.5 (73.8, 75.1)^b^	76.8 (76.2, 77.5)^c^	74.0 (73.1, 74.9)^ab^	<0.001
Sex, *n* (% male)	619 (35)	206 (34)	174 (29)	104 (33)	0.082
BMI, kg/m^2^	27.5 (27.2, 27.7)^a^	28.5 (28.1, 28.9)^b^	27.8 (27.4, 28.2)^ab^	27.9 (27.4, 28.5)^ab^	0.001
Serum total vitamin B-12, pmol/L	268 (262, 274)^a^	263 (252, 273)^a^	268 (257, 279)^a^	229 (215, 243)^b^	<0.001
Serum holoTC, pmol/L	62.7 (61.3, 64.1)^a^	60.0 (57.5, 62.5)^ab^	57.5 (54.9, 60.1)^b^	48.3 (44.9, 51.7)^c^	<0.001
Plasma homocysteine, µmol/L	14.3 (14.0, 14.6)^a^	14.5 (14.1, 15.0)^a^	14.8 (14.3, 15.3)^a^	16.3 (15.7, 17.0)^b^	<0.001
cB12 indicator, *n* (% deficient)^5^	264 (15)	102 (17)	148 (25)	122 (38)	<0.001

1 Data presented are adjusted mean (95% CI) unless otherwise indicated. cB12, combined B12; holoTC, holotranscobalamin; PPI, proton-pump inhibitor.

2 A small number of participants (*n* = 9, 0.7%) could not be classified as regards PPI dose and are not included in this analysis.

3 Participants without atrophic gastritis and nonusers of PPI drugs.

4 *P* < 0.05; analysis via χ^2^ for categorical variables or ANCOVA for continuous variables (adjusted for age, sex, BMI, RBC folate, creatinine, alcohol units per week, fortified food consumption) on log-transformed data where appropriate with Bonferroni post hoc tests. Values across a row without a common superscript letter are significantly different.

5 cB12 indicator was calculated using serum total vitamin B-12, serum holoTC, plasma homocysteine, serum folate, and age to provide a combined indicator value. A value ≤ −0.5 was defined as deficient (20).

**FIGURE 2:**
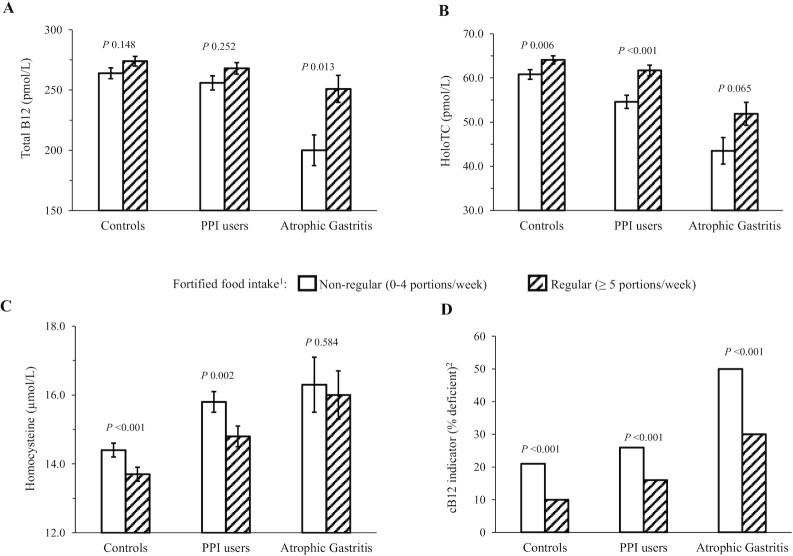
Vitamin B-12 biomarkers and the use of vitamin B-12–fortified food intake in relation to PPI drug use and atrophic gastritis (*n* = 3298). (A) Serum total vitamin B-12; (B) serum holoTC; (C) plasma homocsyteine; and (D) cB12 indicator. Data are presented as adjusted mean (±SEM) or percentage. *P* < 0.05; analysis by χ^2^ for categorical variables or ANCOVA for continuous variables (adjusted for age, sex, BMI, RBC folate, creatinine, alcohol units per week, and PPI dosage in milligrams per day) on log-transformed data where appropriate with Bonferroni post hoc tests. ^1^Participants were classed as nonregular (0–4 portions/wk) or regular (≥5 portions/wk) consumers of vitamin B-12–fortified foods. The most commonly eaten fortified foods were ready-to-eat breakfast cereals (providing added vitamin B-12 levels of 1.6–2.5 µg/100 g; equating to an intake of 0.5–0.8 µg/30-g serving), and to a lesser extent specific brands of fat spreads (providing 2.5–5 µg/100 g; equating to an intake of 0.25–0.5 µg/10-g serving). One participant had missing data for fortified food consumption and could not be classified, and therefore was not included in this analysis. The *n* values for the nonregular groups were: controls, *n* = 797; PPI users, *n* = 518; and atrophic gastritis, *n* = 134. The *n* values for the regular groups were: controls, *n* = 968; PPI users, *n* = 698; and atrophic gastritis, *n* = 183. ^2^cB12 indicator was calculated using serum total vitamin B-12, serum holoTC, plasma homocysteine, serum folate, and age to provide a combined indicator value. A value of ≤ −0.5 was defined as deficient (20). cB12, combined B12; holoTC, holotranscobalamin; PPI, proton-pump inhibitor.

Analysis of the FFQ data showed that the most commonly eaten fortified foods were ready-to-eat breakfast cereals (providing added vitamin B-12 levels of 1.6–2.5 µg/100 g; equating to an intake of 0.5–0.8 µg/30-g serving), and to a lesser extent specific brands of fat spreads (providing 2.5–5 µg/100 g; equating to an intake of 0.25–0.5 µg/10-g serving). The influence of vitamin B-12–fortified food intake on vitamin B-12 biomarkers was examined in the 3 groups. Regular, compared with nonregular, consumption of fortified foods (i.e., ≥5 and 0–4 portions/wk, respectively) impacted positively on all vitamin B-12 biomarkers in all participants and was associated with a significantly lower prevalence of vitamin B-12 deficiency (**Figure 2**). Rates of vitamin B-12 deficiency were up to twice as high in nonregular fortified food consumers in controls, PPI users, and AG groups; the highest prevalence of deficiency (50%) was found in the AG group who did not regularly consume fortified food, and the lowest prevalence (10%) in controls who were regular fortified food consumers (**Table 3**). However, regular consumption of fortified foods (i.e., ≥5 portions/wk) appeared inadequate in normalizing vitamin B-12 status in participants with AG, with 30% vitamin B-12 deficiency found in those regularly consuming these foods (Figure 2; Table 3). When these relations were further explored in males and females separately, similar findings were observed, although in males who were PPI users or had AG, fortified food intakes showed no significant associations with any of the vitamin B-12 biomarkers (**Supplemental Table 1**). There was no difference in age between regular and nonregular fortified food consumers (*P* = 0.741; not shown).

**TABLE 3:** Vitamin B-12 biomarkers in relation to PPI drug usage and atrophic gastritis characterized by vitamin B-12–fortified food intake (*n* = 3298)^1^

	Fortified food consumption^2^							
	Nonregular				Regular			
	*n =* 1449				*n =* 1849			
	Controls^3^	PPI users	Atrophic gastritis		Controls^3^	PPI users	Atrophic gastritis	
	*n =* 797	*n =* 518	*n =* 134	*P* value^4^	*n =* 968	*n =* 698	*n =* 183	*P* value^4^
Age, y	73.0 (72.4, 73.5)^a^	75.8 (75.1, 76.5)^b^	73.1 (71.7, 74.4)^a^	<0.001	72.6 (72.1, 73.2)^a^	75.5 (74.6, 76.1)^b^	74.7 (73.5, 75.8)^b^	<0.001
Sex, *n* (% male)	284 (36)	167 (32)	44 (33)	0.422	334 (35)	216 (31)	60 (33)	0.312
BMI, kg/m^2^	27.7 (27.4, 28.1)	27.7 (27.3, 28.2)	28.3 (27.4, 29.1)	0.619	27.2 (26.9, 27.5)^a^	28.4 (28.1, 28.8)^b^	27.7 (26.9, 28.4)^ab^	<0.001
Serum total vitamin B-12, pmol/L	257 (248, 265)^a^	253 (241, 264)^a^	196 (175, 216)^b^	<0.001	277 (269, 285)^a^	275 (265, 285)^a^	256 (237, 276)^b^	0.022
Serum holoTC, pmol/L	58.3 (56.2, 60.4)^a^	53.5 (50.7, 56.4)^b^	43.1 (38.0, 48.3)^c^	<0.001	66.1 (64.2, 68.0)^a^	62.7 (60.4, 65.1)^b^	52.3 (47.7, 56.8)^c^	<0.001
Plasma homocysteine, µmol/L	15.2 (14.7, 15.6)^a^	15.8 (15.2, 16.4)^a^	17.7 (16.6, 18.8)^b^	0.001	13.6 (13.3, 14.0)^a^	13.8 (13.4, 14.2)^a^	15.3 (14.5, 16.1)^b^	0.003
cB12 indicator, *n* (% deficient)^5^	166 (21)	136 (26)	67 (50)	<0.001	98(10)	115 (16)	55 (30)	<0.001

1 Data presented are adjusted mean (95% CI) unless otherwise indicated. cB12, combined B12; holoTC, holotranscobalamin; PPI, proton-pump inhibitor.

2 Participants were classed as nonregular (0–4 portions/wk) or regular (≥5 portions/wk) consumers of vitamin B-12–fortified foods. The most commonly eaten fortified foods were ready-to-eat breakfast cereals (providing added vitamin B-12 levels of 1.6–2.5 µg/100 g, equating to an intake of 0.5–0.8 µg/30-g serving), and to a lesser extent specific brands of fat spreads (providing 2.5–5 µg/100 g, equating to an intake of 0.25–0.5 µg/10-g serving). One participant had missing data for fortified food consumption and could not be classified, and therefore was not included in this analysis.

3 Participants without atrophic gastritis and nonusers of PPI drugs.

4 *P* < 0.05; analysis via χ^2^ for categorical variables or ANCOVA for continuous variables (adjusted for age, sex, BMI, RBC folate, creatinine, alcohol units per week) on log-transformed data where appropriate with Bonferroni post hoc tests. Values across a row without a common superscript letter are significantly different.

5 cB12 indicator was calculated using serum total vitamin B-12, serum holoTC, plasma homocysteine, serum folate, and age to provide a combined indicator value. A value ≤ −0.5 was defined as deficient (20).

## Discussion

To our knowledge, this is the first study to investigate vitamin B-12 status in relation both to AG and PPI use, and to consider the role of fortified foods. AG, characterized biochemically by a pepsinogen I:II ratio <3, was identified in 15% of the study population and was associated with lower vitamin B-12 status and a greater prevalence of vitamin B-12 deficiency compared with PPI drug users and controls. PPI drugs were used (≥6 mo) by 37% of participants and were adversely associated with vitamin B-12 status, but only in participants taking higher drug doses. Regular consumption of fortified foods (i.e., ≥5 portions/wk) was associated with a lower risk of vitamin B-12 deficiency in all participants and particularly so in those with AG.

The 15% prevalence of AG identified in this study is similar to another community-dwelling study of older adults in Sweden (14%) (11), higher than reported in New Zealand and Germany (7% and 6%, respectively; 7, 28) and considerably lower than the 32% reported in studies undertaken some time ago (8, 9). Pepsinogen analysis, an indirect test to identify AG, was used in this and previous studies; nevertheless, analytical kits and the criteria used to define the condition differ between studies (29, 30). This difference might explain the reported variability in prevalence of this condition to some extent, but demographics, lifestyle, and/or health status of the populations studied could also account for the observed differences. Although AG has been recognized for many years as a contributing factor to vitamin B-12 malabsorption in older people (2), very few studies have determined its association with vitamin B-12 biomarkers. In good agreement with the current findings, the limited evidence from previous reports shows that AG is associated with lower serum total vitamin B-12 (8), higher concentrations of MMA and homocysteine (11, 31), and greater rates of vitamin B-12 deficiency (7, 9, 11). The current study, however, is the first to investigate this relation using multiple vitamin B-12 biomarkers, alone and in combination. Using a cB12 indicator (22), the prevalence of vitamin B-12 deficiency was found to be 2.5-fold higher in those with AG compared with controls. The great variability in rates of vitamin B-12 deficiency reported in previous studies of AG (8, 11) could be attributable to the use of different biomarkers and/or different cutoff values to define deficient vitamin B-12 status. The use (or combination) of ≥2 biomarkers is generally recommended to investigate vitamin B-12 status, given the inherent limitations of each biomarker (21); such an approach would more accurately identify deficiency owing to AG.

In this study, PPI use was associated with significantly lower holoTC concentrations and a higher prevalence of vitamin B-12 deficiency (25% compared with 15% in controls), but only in participants treated with higher PPI doses (≥30 mg/d). In agreement with some previous studies (16, 32), we found no significant relation using serum total vitamin B-12 as the biomarker, whereas others reported that chronic PPI use was associated with lower serum vitamin B-12 concentrations (33) and an estimated 83% increased risk of vitamin B-12 deficiency as found in a meta-analysis (14). Previous studies have also considered the functional indicators, MMA and homocysteine, when examining associations of vitamin B-12 with PPI use, although reporting conflicting findings (17, 18, 32). Discrepant findings in studies regarding the PPI–vitamin B-12 relation could be due to differences in study design, study populations and PPI dose, choice of biomarkers to measure vitamin B-12 status, and/or cutoff points to define deficiency. This is the first study of its kind to measure holoTC, transcobalamin-bound and thus the metabolically active fraction of total vitamin B-12. This is a more recent alternative to total vitamin B-12 concentrations and is generally considered to be a sensitive indicator of status and/or of recent absorption, although this is somewhat controversial (4, 34). The finding that holoTC but not total vitamin B-12 concentrations were significantly associated with PPI use and sufficiently sensitive to detect a dose effect of PPI usage on vitamin B-12 status, suggests that it could be important in future studies to include other biomarkers along with serum total vitamin B-12. In broad agreement with the current findings, in the largest study to date, of >25,000 vitamin B-12–deficient cases and nearly 200,000 controls (identified using an electronic pharmacy database to determine PPI exposure), the risk of a subsequent diagnosis of vitamin B-12 deficiency was shown to be greater at higher PPI treatment levels (i.e., 95% risk of deficiency with >1.5 pills/d compared with a 63% risk with <0.75 pills/d), although PPI dose per se was not reported (19). Both studies, however, highlight the importance of considering PPI dose when investigating associations with vitamin B-12 status. In addition, the impact of duration of PPI use (apart from simply ≥6 mo considered here) is required to give a clear indication of the extent to which this drug, commonly used by older adults, impacts longer-term vitamin B-12 status, and whether monitoring vitamin B-12 status in PPI users should be recommended as routine practice. Prior to any such recommendation, however, the appropriate biomarkers to use and the cutoff values to apply to identify vitamin B-12 deficiency, need to be more fully investigated and validated—issues recently identified among the research gaps in a major vitamin B-12 review by a panel of international experts in vitamin B-12 (1).

Regular compared with nonregular dietary intake of vitamin B-12–fortified foods (i.e., ≥5 and 0–4 portions/wk, respectively) was associated with improved biomarker status and a lower prevalence of deficiency in all participants, whether they had AG or were PPI users or controls. Rates of vitamin B-12 deficiency were found to be up to twice as high in nonregular fortified food consumers in all groups; the highest prevalence of deficiency (50%) was found in the AG group who did not regularly consume fortified food, and the lowest prevalence (10%) in controls who were regular fortified food consumers. Despite the benefits of fortified foods, vitamin B-12 deficiency remained markedly higher in the AG group compared with controls in consumers (30% compared with 10%, respectively), suggesting that current fortification practices provide inadequate levels of vitamin B-12 to normalize vitamin B-12 status in those with AG. In the United Kingdom and Ireland, a voluntary but liberal food fortification policy that includes vitamin B-12 is in place. Evidence shows that such a policy is very effective for enhancing folate, vitamin B-6, and riboflavin status, but less effective for vitamin B-12 status (24, 35–37), and there are reports that the number of products fortified with B vitamins in Ireland has declined over time (38), potentially placing older adults at even greater risk of vitamin B-12 deficiency. The current findings are novel and support the US approach whereby people aged >50 y are recommended to consume the majority of their vitamin B-12 from crystalline sources to overcome food-bound malabsorption (10). Consideration should, however, be given to encouraging food manufacturers to increase vitamin B-12 levels added during fortification and extend the availability of fortified foods to help optimize vitamin B-12 status in older adults at risk of deficiency owing to hypochlorhydria.

Vitamin B-12 deficiency is a global problem, associated with several adverse health outcomes (1, 6). Poor dietary intake is considered a major cause of deficiency in low- and middle-income countries, typically in regions where vegan diets or limited animal foods are consumed, but gastrointestinal infections and infestations, along with host–microbiota interactions, can also be contributory factors (4). The deficient vitamin B-12 status commonly found in older adults in high-income countries is, however, rarely attributable to low dietary intakes, which are typically found to exceed current recommendations (39), but rather the result of malabsorption owing to hypochlorhydria. As shown in the current study, older adults with AG or taking PPI drugs can thus benefit from consuming fortified foods, given that the crystalline vitamin B-12 form added in the fortification process—unlike protein-bound vitamin B-12 in natural food sources—does not require gastric acid for its absorption. Furthermore, reported vitamin B-12 supplement use in older adults is much lower in Europe (<11%; 37, 40) than in the United States (36%; 41), potentially placing older European adults at greater risk of vitamin B-12 deficiency. In order to protect older adults from potentially higher risk of cognitive dysfunction, osteoporosis, and cardiovascular disease associated with low vitamin B-12 status (6), further research investigating the efficacy of interventions to improve vitamin B-12 status is required. Also, in future studies ≥2 vitamin B-12 biomarkers should be used, and ideally the combined vitamin B-12 indicator calculated, to overcome the limitations of individual biomarkers (20, 22).

The major strengths of this study were the availability of a large and well-characterized cohort of older adults, and use of both direct and indirect biomarkers separately and in combination, to assess vitamin B-12 status, an approach recommended by experts in the field (20, 22). Additionally, it is the first study to explore the potential of fortified foods to protect against any depletion in vitamin B-12 status that might arise as a result of hypochlorhydria. The study is, however, not without limitations. Because the study was observational in design, causal relations between PPI use or AG and vitamin B-12 deficiency cannot be inferred. Also, although PPI dosage was recorded, information on duration of use was limited to <6 or ≥6 mo. Furthermore, the pepsinogen I:II ratio is not a direct method for diagnosing AG; however, it has high specificity and is considered a reliable noninvasive screening tool for this condition (30).

In conclusion, older adults who have AG and/or higher-dose PPI use are more likely to have indicators of vitamin B-12 deficiency. Fortified foods, if consumed regularly, are associated with enhanced vitamin B-12 status, but higher levels of added vitamin B-12 than currently provided could be warranted to optimize status in older adults at greatest risk of food-bound malabsorption related to hypochlorhydria. Research is warranted to investigate further the relations of AG and PPI use with vitamin B-12 status in older age.

## Supplementary Material

nqab193_Supplemental_File

## Data Availability

Data described in the manuscript, code book, and analytic code will be made available upon request pending application and approval.

## Notes

Supported by governmental funding from the Irish Department of Agriculture, Food and the Marine and Health Research Board (under the Food Institutional Research Measure initiative) and the Northern Ireland Department for Employment and Learning (under its Strengthening the All-Island Research Base initiative).

Supplemental Table 1 is available from the “Supplementary data” link in the online posting of the article and from the same link in the online table of contents at https://academic.oup.com/ajcn/.

KMP and LH Joint first authors.

Abbreviations used: AG, atrophic gastritis; cB12, combined B12; holoTC, holotranscobalamin; MMA, methylmalonic acid; PPI, proton pump inhibitors; TUDA, Trinity-Ulster and Department of Agriculture.
