# Supplementary material for: Associations of atrophic gastritis and proton-pump inhibitor drug use with vitamin B-12 status, and the impact of fortified foods, in older adults
Source: Am J Clin Nutr. 2021 Jun 16;114(4):1286–94. doi: 10.1093/ajcn/nqab193 (PMC8488868; doi:10.1093/ajcn/nqab193)
Supplement: nqab193_Supplemental_File [file nqab193_supplemental_file.docx]

**Supplementary Table 1** Vitamin B12 biomarkers in relation to PPI drug usage and atrophic gastritis characterised by B12 fortified food intake in males and females (n = 3298)^1^

|  | Males  *n* = 1105 | | | | | |
| --- | --- | --- | --- | --- | --- | --- |
|  | Controls | | PPI users | | Atrophic Gastritis | |
|  | Non-regular  *n* = 284 | Regular *n* = 334 | Non-regular  *n* = 167 | Regular *n* = 216 | Non-regular  *n* = 44 | Regular *n* = 60 |
| Age (years) | 71.4 (70.3, 72.4) | 72.0 (71.0, 73.0) | 74.2 (72.7, 75.8) | 73.5 (72.2, 74.8) | 72.7 (69.3, 76.1) | 73.5 (70.5, 76.5) |
| BMI (kg/m^2^) | 28.2 (27.6, 28.8) | 28.4 (27.9, 29.0) | 27.6 (26.6, 28.5) | 28.5 (27.7, 29.3) | 28.5 (26.8, 30.1) | 29.0 (27.5, 30.5) |
| Serum total B12 (pmol/L) | 243 (230, 256) | 260 (248, 272) | 254 (236, 271) | 252 (237, 266) | 219 (174, 264) | 212 (172, 252) |
| Serum holoTC (pmol/L) | 54.2 (51.2, 57.1) | 60.5 (57.8, 63.3)^*^ | 50.4 (46.1, 54.8) | 55.4 (51.8, 59.0) | 47.6 (37.4, 57.7) | 46.7 (37.6, 55.8) |
| Plasma homocysteine (µmol/L) | 14.9 (14.4, 15.3) | 14.2 (13.8, 14.6)^*^ | 16.7 (15.6, 17.8) | 15.9 (15.0, 16.8) | 18.1 (15.0, 21.2) | 16.6 (13.9, 19.3) |
| cB12 indicator, *n* (% deficient)^4^ | 68 (24) | 37 (11)^*^ | 48 (29) | 42 (19)^*^ | 22 (50) | 19 (32) |

|  | Females *n* = 2193 | | | | | |
| --- | --- | --- | --- | --- | --- | --- |
|  | Controls | | PPI users | | Atrophic Gastritis | |
|  | Non-regular  *n* = 513 | Regular *n* = 634 | Non-regular  *n* = 351 | Regular *n* = 482 | Non-regular  *n* = 90 | Regular *n* = 123 |
| Age (years) | 72.1 (71.2, 72.9) | 71.5 (70.7, 72.2) | 73.8 (72.5, 75.1) | 74.0 (72.9, 75.0) | 71.5 (69.5, 73.5) | 73.6 (71.9, 75.4) |
| BMI (kg/m^2^) | 26.9 (26.2, 27.5) | 26.1, 25.6, 26.6) | 28.2 (27.3, 29.1) | 28.8 (28.1, 29.5) | 28.2 (26.4, 30.0) | 27.6 (26.0, 29.2) |
| Serum total B12 (pmol/L) | 278 (265, 289) | 283 (272, 293) | 259 (245, 275) | 277 (265, 290) | 191 (161, 221) | 273 (246, 299) |
| Serum holoTC (pmol/L) | 65.1 (62.2, 67.9) | 66.4 (64.0, 68.9) | 57.6 (53.7, 61.4) | 65.5 (62.3, 68.6)^*^ | 41.3 (34.1, 48.5) | 54.6 (48.2, 61.0)^*^ |
| Plasma homocysteine (µmol/L) | 14.1 (13.6, 14.6) | 13.4 (13.0, 13.8)^*^ | 15.3 (14.6, 16.1) | 14.2 (13.6, 14.8)^*^ | 15.3 (13.7, 16.8) | 15.7 (14.3, 17.1) |
| cB12 indicator, *n* (% deficient)^4^ | 98 (19) | 61 (10)^*^ | 88 (25) | 73 (15)^*^ | 45 (50) | 36 (30)^*^ |

^1^Data presented are adjusted mean (95% CI) unless otherwise indicated. cB12, combined B12; holoTC, holotranscobalamin; PPI, proton-pump inhibitor.

^2^Analysis via chi-square for categorical variables or ANCOVA for continuous variables (adjusted for age, BMI, red blood cell folate, creatinine, alcohol units/week) on log transformed data where appropriate with Bonferroni post-hoc tests. ^*^Denotes significance between fortified food intake within each group; *P* <0.05.

^3^Participants were classed as non-regular (0-4 portions/week) or regular (≥ 5 portions/week) consumers of B12 fortified foods. The most commonly eaten fortified foods were ready-to-eat breakfast cereals (providing added B12 levels of 1.6- 2.5 µg/100g; equating with an intake of 0.5 to 0.8 µg/30g serving), and to a lesser extent specific brands of fat spreads (providing 2.5-5 µg/100g; equating with an intake of 0.25 to 0.5 µg/10g serving). One participant had missing data for fortified food consumption and could not be classified, and therefore was not included in this analysis.

^4^cB12 indicator was calculated using serum total vitamin B12, serum holoTC, plasma homocysteine, serum folate and age to provide a combined indicator value. A value of ≤ -0.5 was defined as deficient (20).
